# Digital communication symbols and intergenerational gap: a case study analysis of Netflix’s “Adolescence”

**DOI:** 10.3389/fsoc.2026.1633629

**Published:** 2026-03-19

**Authors:** Vinanda Cinta Cendekia Putri, Muh. Akbar, Irwanto Irwanto, Sonni Alem Febri

**Affiliations:** 1 Digital Communication Study Program, Faculty of Vocational Studies, Hasanuddin University, Makassar, Indonesia; 2 Communication Sciences, Faculty of Social and Political Sciences, Hasanuddin University, Makassar, Indonesia; 3 Film Department, School of Design, Bina Nusantara University, Jakarta, Indonesia

**Keywords:** digital communication, digital symbols, emoji, generation alpha, generation Z, intergenerational communication, media literacy

## Abstract

Generational differences in interpreting digital communication symbols, including emoji, slang, and memes, create significant communication barriers between Generation Z/Alpha and older generations, with potentially serious social and legal consequences. This study examines how such gaps produce blind spots in which concerning online behaviors, including cyberbullying, can develop undetected by adult authority figures. Using Netflix’s limited series Adolescence (2025) as a case study, this research employs qualitative content analysis of the film’s portrayal of digital communication alongside quantitative analysis of social media discussions about the series to identify patterns of symbolic misinterpretation across generational lines.Three key areas of intergenerational misinterpretation were identified: context-dependent emoji usage, rapidly evolving digital slang, and generation-specific cultural references embedded in memes. These communicative gaps systematically prevent adult authority figures from recognizing warning signs of concerning online behavior, reduce opportunities for timely intervention, and risk the misinterpretation of digital evidence in legal contexts. This study contributes to digital literacy literature by characterizing the specific mechanisms through which intergenerational communication failures occur. By mapping these communicative spaces, it establishes an empirical and theoretical foundation for developing targeted educational interventions in family and institutional settings, as well as informing future prevention strategies for online risk behaviors.

## 1 Introduction

Digital communication technologies, including social media platforms (e.g., Facebook, Instagram, TikTok, X/Twitter), instant messaging applications (e.g., WhatsApp, Telegram), and video-sharing services (e.g., YouTube, Snapchat), have transformed human interactions, creating new symbolic languages that evolve quickly. According to recent data, over 5.04 billion people worldwide use social media as of 2024, representing 62.3% of the global population, and spend an average of 2 h and 23 min daily on these platforms (DataReportal, 2024). These communication modalities, from emoji to internet slang and memes, evolve so rapidly that they often create significant comprehension gaps between generations (Jones and Hafner, 2021).

Netflix’s limited series “Adolescence” (2025), directed by Jack Thorne and starring Stephen Graham, offers a compelling cultural artifact for examining these intergenerational communication issues. The narrative centers on Jamie Miller, a 13-year-old arrested for the murder of a female classmate after being radicalized in online communities without his parents’ knowledge or understanding. The series explicitly addresses how generational differences in comprehending digital communication can have profound consequences, serving as both a dramatic representation and a cautionary tale about these divides.

As noted in a Guardian review, the series highlights “the stark gap between parents’ image of knowing nothing about their children’s lives and the truth about what they do online” (Hogan, 2025). This observation encapsulates the phenomenon this study seeks to explore: the gap between how younger generations with extensive digital media exposure (Generation Z, born 1997–2010, and Generation Alpha, born 2010–2025) engage with digital communication symbols and how these same symbols are interpreted by older generations (Millennials, Generation X, and Baby Boomers). While terms like “digital natives” have been widely used to describe these younger cohorts, scholars have critically questioned the validity of this concept, noting that it oversimplifies the complex relationships among age, technology access, and digital competence (Bennett et al., 2008; Helsper and Eynon, 2009). Rather than assuming innate generational differences, this study examines observed patterns in how different age groups interpret and assign meaning to digital symbols, recognizing that these differences emerge from varying contexts of exposure, socialization, and media consumption practices rather than inherent technological affinity.

This research employs “Adolescence” as a case study to analyze how intergenerational differences in the interpretation of digital communication symbols emerge in the series’ narrative and public discourse. By examining the portrayal of digital communication in the series alongside real-world conversations about the show, this study aims to identify patterns of misinterpretation, their potential consequences, and possible strategies for bridging these communicative divides.

### 1.1 Scope and contribution of this research

It is important to clarify the scope and contribution of this study at the outset. This research neither proposes nor evaluates specific cyberbullying prevention interventions. Rather, it provides a foundational analysis that identifies the communicative mechanisms and interpretive gaps through which concerning online behaviors, including but not limited to cyberbullying, can develop without detection by parents, educators, or other authority figures. Our contribution is descriptive and analytical rather than prescriptive: we systematically map the “blind spots” in intergenerational digital communication to illuminate where and how misinterpretations occur, characterize the patterns of these misunderstandings, and document their potential consequences. This foundational work establishes the basis for designing and targeting future prevention efforts. By identifying the specific communicative spaces in which problems can develop undetected and characterizing the mechanisms by which digital symbols are misinterpreted across generations, we provide essential groundwork for subsequent intervention development and testing. As the Chief Specialty Editor, Scott has noted, we are “identifying those spaces within which prevention might profitably occur” rather than proposing the prevention mechanisms themselves.

### 1.2 Digital communication across generations

Research consistently demonstrates significant differences in how various generations approach digital communication. Generation Z (born approximately 1997–2012) represents a complex generational cohort in terms of digital nativity. While the concept of “digital natives” was initially proposed Prensky (2001) to describe those who grew up immersed in digital technology from birth, recent critical scholarship has challenged this monolithic categorization (Bennett et al., 2008). Generation Z’s relationship with digital technology varies significantly depending on their birth year within the cohort: older Gen Z members (born 1997–2005) experienced substantial childhood years with pre-smartphone technology and witnessed the evolution from Web 1.0 to Web 2.0, while younger Gen Z members (born 2006–2012) have had access to smartphones and social media from early childhood. This cohort demonstrates proficiency with multiple social media platforms but, unlike Generation Alpha, still maintains significant value for face-to-face interaction and experienced formative years with both analog and digital communication modalities (Chen and Ha, 2023; Prensky, 2001; Bennett et al., 2008). They typically engage with longer text formats, employ hashtags strategically, and were shaped by early digital culture phenomena like Vine and YouTube (Fitri, 2024).

In contrast, Generation Alpha has never known a world without mature digital technologies. Their communication patterns tend toward brevity, visuals, and immediacy characteristics fostered by their primary platforms: TikTok, YouTube Shorts, and Instagram Reels. Their linguistic capabilities reflect global access to information and rapid content consumption, with a preference for concise, visual communication that incorporates emoji, GIFs, and video effects (Fitri, 2024; Putri et al., 2025).

Older generations, particularly Generation X and Baby Boomers, typically view digital communication technologies primarily as functional tools rather than as integrated social spaces. Their communication style often relies on literal interpretation of text and conventional understanding of digital symbols, lacking familiarity with the contextual and rapidly evolving meanings prevalent among younger users (Capobianco, 2022; Howry et al., 2023).

### 1.3 Digital symbols and their interpretation

The literature identifies three key categories of digital symbols where intergenerational misunderstandings frequently occur: emoji, slang expressions, and memes.

Emoji interpretation varies significantly across generations. Research indicates that approximately 70% of Generation Z users use emojis in ways that deviate from their literal meanings, often ironically or as in-group humor markers (Mutual, Physicians, 2023). For example, the skull emoji (💀) frequently signifies extreme amusement among younger users, equivalent to “I am dead [from laughter]”. At the same time, older generations may interpret it literally as a representation of death or danger (Shoichet, 2024). Similarly, the thumbs-up emoji (👍) often carries connotations of passive aggression or sarcasm for Gen Z but represents sincere approval for older users (Mihăilescu, 2024).

Digital slang evolves rapidly, with expressions gaining and losing currency within months. Terms popular among Generation Z (“stop lying,” “bad vibes”) differ from those used by Generation Alpha (“sigma,” “stop the cap,” “ate,” “yapping”), creating potential comprehension barriers not only with older generations but even between these adjacent young cohorts (Fitri, 2024). X discussions reflected this awareness, with one user noting: “My teenage daughter says I ‘ate’ when I do something well, but my 10-year-old son uses completely different terms. I can barely keep up with one generation, let alone both!” (illustrating inter-generational slang gaps within families). Another post observed: “Teachers are struggling because Gen Z slang is already outdated—Gen Alpha kids are using ‘sigma,’ ‘rizz,’ and ‘gyat,’ which sound like complete nonsense to anyone over 25” (highlighting educational communication barriers). The speed at which this linguistic evolution occurs makes it nearly impossible for non-immersed users to maintain their current understanding (McCulloch, 2022).

Memes represent an even more complex form of digital communication, combining images and text to convey meaning that relies heavily on shared cultural references and contextual knowledge. The absurdist and ironic nature of contemporary memes makes them particularly challenging for audiences not deeply engaged with specific online communities or current digital culture (Shifman, 2023). X users frequently commented on this comprehension gap. One parent posted: “I found a meme on my son’s phone that looked disturbing (Pepe frog with weird text), but he said it was just ‘ironic humor.’ How am I supposed to know what’s concerning?” (expressing parental confusion about meme interpretation). Another educator shared: “A student submitted a project full of memes I didn’t understand. Turns out they were all relevant and clever, but I almost marked it down because I thought they weren’t taking it seriously” (illustrating institutional misunderstanding). A third post noted: “The series perfectly captured how what looks like extremist content to parents might just be edgy memes to kids—but also how actual extremist content hides behind ‘ironic’ memes” (acknowledging the genuine ambiguity in meme interpretation).

Memes represent the most complex form of digital communication, combining images and text to convey ideas that depend heavily on shared cultural references and contextual knowledge. For instance, the “Distracted Boyfriend” meme format uses a stock photo of a man looking at another woman. In contrast, his girlfriend looks on disapprovingly, but when captioned, can represent countless scenarios of temptation or divided attention. Similarly, the “Woman Yelling at Cat” meme juxtaposes an image from “The Real Housewives of Beverly Hills” with a confused-looking cat at a dinner table, creating a versatile format for depicting arguments or disagreements (Anderau and Barbarrusa, 2024).

In the context of “Adolescence,” memes referencing manosphere ideologies or incel culture would carry coded meanings recognizable to those familiar with these online communities but appear as innocuous humor to uninformed observers. Recent research has demonstrated how memes function not merely as entertainment but as vehicles for political discourse and identity formation, with harmful memes playing documented roles in radicalization processes (Grasso et al., 2024). The layered, often ironic or absurdist nature of contemporary memes makes them particularly challenging for those not regularly engaged with specific online communities or current digital culture (Shifman, 2023; Tidy et al., 2024).

### 1.4 Digital communication misunderstandings and their consequences

The literature suggests that misinterpreting digital communication symbols can have serious consequences across multiple domains. In family relationships, parents’ inability to comprehend their children’s digital communication can erode trust and prevent practical guidance (Livingstone and Blum-Ross, 2023). The quality of parent–child communication is particularly critical in the digital age, as it serves as a protective factor against online risks, including cyberbullying, exposure to inappropriate content, and contact with malicious actors (Tammisalo and Rotkirch, 2022). Research demonstrates that high-quality parent–child communication about digital media use, characterized by openness, active listening, and mutual respect rather than purely restrictive approaches, is associated with better adolescent digital wellbeing and a reduced likelihood of problematic internet use (Tammisalo and Rotkirch, 2022).

This communication quality becomes especially important during adolescence, a developmental period characterized by identity formation and increased peer influence, when young people are simultaneously seeking autonomy and requiring guidance. When parents cannot understand or meaningfully engage with their children’s digital communication practices, several negative consequences may follow: adolescents may become less likely to seek parental guidance when encountering online problems, parents miss opportunities to provide developmentally appropriate scaffolding for digital literacy skills, and mutual mistrust may develop as adolescents perceive parental interest as surveillance rather than supportive engagement (Livingstone and Blum-Ross, 2023; Tammisalo and Rotkirch, 2022).

Empirical research documents specific instances where parent–child digital communication failures have led to serious outcomes. Livingstone and Blum-Ross (2023) Describe cases where parents’ misinterpretation of online communication led to either over-restrictive responses that damaged trust or under-responses that failed to address genuine risks. In one documented case, a parent interpreted a child’s increased use of the crying-laughing emoji (😂) as an indicator of happiness, not recognizing it could signal ironic detachment or mask distress. This misreading delayed recognition of the child’s cyberbullying victimization. Another case involved parents who dismissed their teenager’s references to “edgy memes” as typical adolescent humor, not recognizing that these were entry points to extremist ideological content, similar to the scenario portrayed in “Adolescence” (Livingstone and Blum-Ross, 2023).

While this research does not test specific prevention strategies, our systematic identification of the precise nature of these communicative gaps, the mechanisms through which digital symbols are misinterpreted, and the patterns by which concerning content is rendered invisible to adult observers, provides essential groundwork for developing targeted interventions. Understanding not just that misinterpretation occurs, but how it occurs, what specific symbols are most frequently misunderstood, and what consequences follow from these misunderstandings, establishes the empirical foundation necessary for designing prevention programs that address these specific areas of vulnerability in intergenerational digital communication.

The literature suggests that misinterpreting digital communication symbols can have serious consequences across multiple domains. Parents’ inability to comprehend their children’s digital communication can erode trust and prevent practical guidance in family relationships (Livingstone and Blum-Ross, 2023). In educational settings, these misunderstandings may lead to disciplinary actions based on misinterpreted content or failure to recognize genuine issues (O'Keeffe and Clarke-Pearson, 2021).

Perhaps most concerning are the implications in contexts involving potential harm. Research indicates that adults often miss warning signs of problematic online behavior, mental health crises, or radicalization when these signals are embedded in digital communication that they cannot accurately interpret (Capobianco, 2022). In legal proceedings, misinterpretation of digital evidence, particularly emoji and slang, has influenced case outcomes, prompting some jurisdictions to develop specialized training for legal professionals (Howry et al., 2023).

In legal proceedings, misinterpretation of digital evidence, particularly emoji and slang, has influenced case outcomes, prompting some jurisdictions to develop specialized training for legal professionals (Howry et al., 2023). Several documented cases illustrate the severity of these issues:

In a prominent 2017 case from Israel (Dahan vs. Rosen), the Herzliya Small Claims Court confronted the question of whether emojis could demonstrate intent and good faith in contract negotiations. A prospective tenant sent text messages to a landlord containing multiple enthusiastic emojis, including a smiley face (😊), dancing woman (💃), champagne bottle (🍾), and comet (☄), expressing interest in renting an apartment and stating, “just need to discuss the details.” In reliance on these positive messages, the landlord removed the property from the rental market and began preparing a lease agreement. When the prospective tenants subsequently ceased all communication and rented a different apartment, the landlord sued for damages. Judge Amir Weizebbluth ruled that while the emoji-laden messages did not create a binding contract, they “conveyed great optimism” that reasonably led to the landlord’s reliance on the defendants’ expressed desire to rent. The court awarded approximately $2,200 in damages, concluding the prospective tenants had negotiated in bad faith (Goldman, 2018; Kirley and McMahon, 2017). Though from an Israeli court, this case is extensively cited in U.S. legal scholarship and demonstrates how emoji interpretation can have tangible legal consequences.

More recently, courts in common law jurisdictions have reached differing conclusions about emojis in contractual contexts. In the 2022 New York case Lightstone vs. Zinntex, a court determined that a thumbs-up emoji (👍) in a text conversation did not constitute clear acceptance of contract terms, particularly given that earlier in the conversation the same party had stated he would not sign any contract. Conversely, in the 2023 Canadian case Southwest Terminal Ltd. vs. Achter Land & Cattle Ltd., a Saskatchewan court held that a farmer’s thumbs-up emoji response to a photo of a contract did constitute acceptance and created a valid, binding agreement to sell grain despite the farmer’s subsequent claim that he intended only to acknowledge receipt of the message. These contrasting rulings highlight the ongoing legal uncertainty surrounding emoji interpretation and the contextual nature of determining their communicative intent (Rubin, 2023).

In criminal contexts, misinterpretation carries even higher stakes. Several terrorism-related cases in the United States and Europe have involved prosecutors presenting social media posts containing weapon emojis (🔫), explosion symbols (💣), or knife images (🔪) as evidence of intent or threat. At the same time, defense attorneys have argued these represented common expressive language among young people without literal meaning. The different interpretations of identical emoji sequences by prosecutors (literal threat), defense attorneys (ironic expression), and juries (uncertain) demonstrate the profound interpretive challenges these cases present and the potential for serious consequences stemming from generational or cultural misunderstanding of digital communication norms (Goldman, 2018).

In a case particularly relevant to the themes of “Adolescence,” UK prosecutors in 2019 presented evidence that a defendant charged with planning violence had used specific Pepe the Frog meme variations that, according to expert testimony, functioned as signals of affiliation with extremist ideologies. These memes would have appeared to uninformed observers as random internet humor, but forensic analysis of their specific variations and context of use established their meaning within extremist online communities (Hodge and Hallgrimsdottir, 2019). This case demonstrates how meme literacy and understanding of digital subcultures can be essential for accurate threat assessment and how misinterpretation by authority figures unfamiliar with these communication patterns could result in either failure to recognize genuine dangers or wrongful prosecution based on misunderstood expression.

## 2 Materials and methods

This research employs a mixed-methods approach combining qualitative content analysis of the Netflix series “Adolescence” with quantitative and qualitative analysis of X discussions about the series. This methodological choice was informed by the need to examine the representational aspects of intergenerational digital communication gaps and the public discourse surrounding these issues in response to the cultural artifact.

### 2.1 Qualitative content analysis

We conducted a detailed qualitative content analysis of all seven episodes of “Adolescence,” with particular attention to scenes depicting digital communication, online interactions, and instances where intergenerational misunderstandings of digital symbols played a narrative role. Two researchers independently analyzed each episode, documenting all relevant scenes and reaching consensus on categorization through discussing any initially divergent interpretations.

The analysis employed a systematic coding framework that categorized instances according to three primary dimensions. First, we identified the type of digital symbol involved, including emoji usage, slang expressions, memes, and platform-specific communication norms. Second, we analyzed the nature of the misunderstanding, distinguishing between literal versus contextual interpretations and generational differences in symbol comprehension. Third, we documented the consequences of each misunderstanding within the narrative, tracking how these communication failures contributed to plot development and character relationships.

This framework enabled us to comprehensively map how the series portrays intergenerational digital communication challenges and their narrative implications. By analyzing patterns across these instances, we could identify recurring themes and representational strategies within the series’ treatment of these issues.

### 2.2 X data collection and analysis

To examine real-world discussions of intergenerational digital communication gaps about “Adolescence,” we collected and analyzed posts from the application X (formerly Twitter) about the series that specifically referenced its digital communication aspects. We focused exclusively on X due to its prominence as a platform for public discourse about media content and its accessibility for research purposes compared to more closed platforms. This choice also allowed for a deeper analysis of a single platform’s discourse patterns rather than a more superficial examination across multiple platforms.

Our exclusive focus on X (formerly Twitter) for social media data collection requires acknowledgment of the platform’s particular characteristics and limitations. X has been documented as a significant space for creating and perpetuating extremism, with research demonstrating that the platform’s architecture and moderation policies have facilitated the spread of extremist content, particularly following changes in platform ownership and content moderation approaches in late 2022 (Sengul, 2025). Sengul (2025) Analysis of far-right communities’ use of X Spaces (the platform’s audio conversation feature) reveals how these communities leverage the platform for discourse, communication, and sharing of extremist ideologies. This context is directly relevant to our study, as “Adolescence” depicts similar dynamics of online radicalization through digital platforms. The platform’s role in facilitating extremist discourse means that discussions about the series on X may reflect both awareness of these issues and, potentially, defensive reactions from users engaged in similar online communities to those depicted in the series. This limitation should be considered when interpreting our findings, as the discourse patterns observed on X may differ from those on platforms with different user demographics, content moderation policies, and architectural affordances.

Identifying generational affiliation on X presented methodological challenges, as users do not consistently state their age or generation in their profiles. We employed several indicators where available: (1) explicit age or graduation year statements in user biographies, (2) contextual clues such as references to “my children” or “when I was in school during”, (3) profile images appearing to show users of particular age ranges (though we recognize the limitations of visual age assessment), and (4) language patterns and cultural references suggesting generational cohort. However, we acknowledge that generational identity often remained uncertain, which is reflected in our reporting, where we note user demographics as “when identifiable” rather than claiming comprehensive demographic data.

Our data collection employed carefully refined search parameters to ensure relevance and comprehensiveness. The primary search query combined “Adolescence Netflix” with terms related to digital communication (“digital communication,” “Gen Z language,” “emoji,” “meme,” “generation gap,” “digital slang”). We implemented this search in both English and multiple other languages to capture global discourse, with particular attention to Indonesian-language discussions, given the significant engagement with the series in this region. The data collection period spanned 3 months following the series release (March–May 2025), capturing immediate reactions and more developed analyses that emerged as viewership spread.

We manually filtered an initial dataset of approximately 120 posts matching our search parameters to exclude irrelevant content. This resulted in a final corpus of 20 posts that substantively engaged with the themes of digital communication in “Adolescence.” While this sample size is moderate, it represents the most relevant publicly available discussions of these specific themes about the series.

Our analysis of this X corpus employed both computational and interpretive methods. Two researchers systematically coded each post across multiple dimensions and then resolved any coding discrepancies through discussion. The coding framework included the primary topic addressed (categorized into themes such as educational value, portrayal of online dynamics, parental responsibilities, etc.), explicit mentions of specific digital symbols (emoji, slang terms, memes), sentiment orientation (positive, negative, or neutral toward the issues portrayed), and user demographics when identifiable (including geographic location and, where possible, indicators of generational identity).

Each post was coded for thematic content (e.g., character analysis, plot discussion, parenting responsibilities, etc.), explicit mentions of specific digital symbols (emoji, slang terms, memes), sentiment orientation (positive, negative, or neutral toward the issues portrayed), and user demographics when identifiable (including geographic location, age indicators, and other markers of generational identity).

We employed the VADER (Valence Aware Dictionary and sEntiment Reasoner) tool (Hutto and Gilbert, 2014) for sentiment analysis. VADER is particularly well-suited for social media content analysis due to its sensitivity to intensity modifiers and contextual polarity shifts, which are standard in social media expression. The tool was specifically designed to handle social media-specific linguistic features, including emoticons, slang, acronyms, capitalization for emphasis, and punctuation patterns that convey sentiment intensity (Hutto and Gilbert, 2014). This computational analysis was supplemented by manual close reading to capture nuances in emotional valence that automated tools might miss, particularly in multilingual content and posts that employ irony, sarcasm, or culturally specific references, which lexicon-based sentiment analyzers may not accurately interpret.

Combining these methodological approaches, a detailed content analysis of the series itself, and a systematic examination of X discourse on digital communication themes allowed us to explore the representational strategies employed in “Adolescence” and their reception and interpretation in public discourse. This dual focus provides insight into how intergenerational digital communication gaps are portrayed and how these portrayals resonate with and stimulate discussion about real-world communication challenges across generations.

## 3 Results

### 3.1 Representation of digital communication in “adolescence”

Comprehensive content analysis of “Adolescence” identified 37 significant scenes depicting digital communication across the seven-episode series, of which 18 (48.6%) portray generational misunderstandings. These scenes were not evenly distributed across episodes but concentrated in episodes three through five, suggesting an intentional narrative arc that builds toward the central crisis of the story.

In one particularly significant scene in episode four, Jamie’s use of the skull emoji (💀) within messages about an online forum created a layered misunderstanding. His parents, aware that young people use this emoji to express amusement, interpreted Jamie’s messages as indicating he found the forum content funny in a lighthearted way ‘just internet humor.’ However, while the parents correctly identified that Jamie was using the emoji to express an extreme positive reaction, they fundamentally misunderstood what he was reacting to so positively. The skull emoji was indeed expressing “I’m dead [this is so good],” but it signified Jamie’s genuine appreciation and excitement for violent and misogynistic content rather than innocuous humor. The tragedy lay not in misunderstanding the emoji’s function, but in the parents’ failure to critically examine what content was eliciting such enthusiastic responses from their son. This represents a more subtle form of digital communication failure: understanding the symbolic language while missing its concerning application.

The second most common category involved authority figures misinterpreting slang in Jamie’s online communications, which occurred in six scenes. These instances primarily featured teachers and later police investigators who lacked familiarity with the specialized vocabulary of the online communities Jamie participated in. In episode five, an investigator reviewing Jamie’s messages failed to recognize that terms like “based,” “redpilled,” and “normie” functioned as in-group markers for a misogynistic online community, interpreting them instead as typical teenage slang without ideological significance. This misinterpretation affected the initial assessment of Jamie’s motivations and delayed recognition of his radicalization pathway.

The third significant category in the five scenes is parents failing to recognize the significance of memes shared on Jamie’s social media that referenced concerning ideologies. These scenes portrayed parents scrolling past or even laughing at memes whose visual language and references encoded ideas from extremist communities. The parents interpreted these as random internet humor, while missing their role as entry points into problematic ideological spaces. This pattern culminated in a scene in episode six where, during a retrospective review of Jamie’s online activity, the parents finally understood the significance of memes they had previously dismissed, illustrating their belated recognition of warning signs they had missed.

Across all these categories, the narrative explicitly establishes a causal connection between these digital communication misunderstandings and the story’s tragic outcome. The series employed both dialogue (characters explicitly discussing communication failures) and narrative structure (juxtaposing scenes of misunderstanding with scenes showing the consequences) to emphasize that the parents’ and authorities’ inability to accurately interpret Jamie’s digital communication directly contributed to their failure to recognize his radicalization and intervene before violence occurred.

### 3.2 X discussions

X data analysis revealed meaningful engagement with the themes of intergenerational digital communication gaps portrayed in “Adolescence.” The dataset of 20 posts that substantially addressed these themes exhibited several significant patterns regarding sentiment, geographic distribution, engagement metrics, and thematic focus.

**Figure 1:**
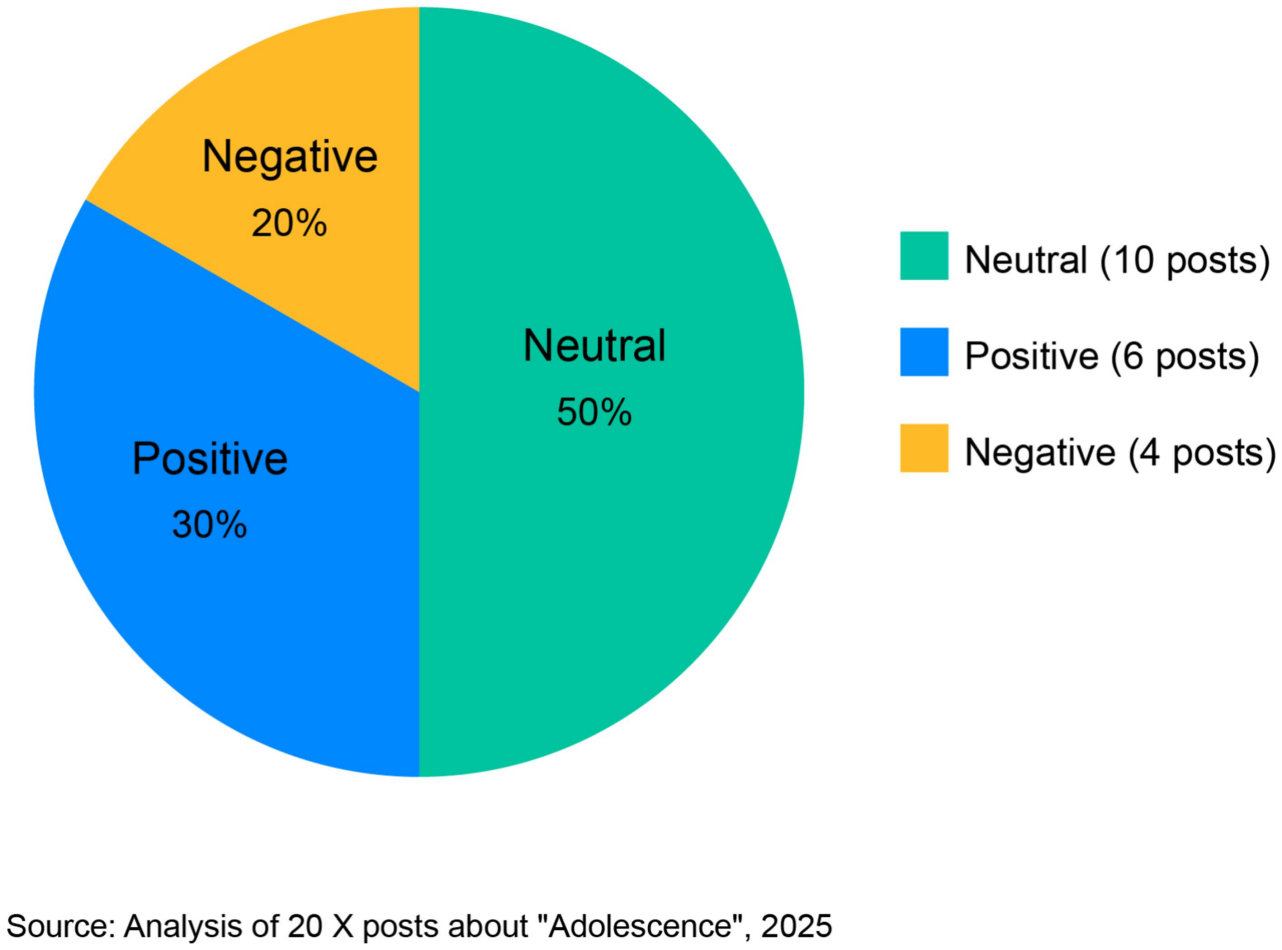
Sentiment distribution in X discussions about “Adolescence.”

As shown in Figure 1, sentiment analysis of the X posts demonstrated a relatively balanced emotional valence in discussions of the series’ digital communication themes. Half of all analyzed posts (10) maintained a neutral tone, presenting observations or analyses without intense emotional coloring. For example, one neutral post stated: “The show accurately depicts how parents completely miss context clues in their kids’ online language. The skull emoji scene was painfully realistic” (coded as neutral analytical observation). Six posts (30%) expressed positive sentiment, praising the series for its educational value or accurate portrayal of intergenerational communication challenges. A representative positive post noted: “Finally, a series that shows parents what we’ve been trying to explain about digital communication! Every parent should watch this” (expressing approval and educational endorsement). The remaining four posts (20%) conveyed negative sentiment, though notably this negativity primarily reflected rising concern about the issues portrayed rather than criticism of the series itself. One negative post expressed: “Watching this makes me terrified about what my kids might be involved in online that I can’t understand. How are we supposed to keep them safe?” (expressing parental anxiety about digital literacy gaps). This distribution suggests that discussions of the series’ digital communication themes were predominantly substantive and reflective rather than purely emotional, with viewers engaging in thoughtful consideration of the issues rather than merely reactive responses.

The geographic distribution of these X discussions, presented in Table 1, demonstrates the global relevance of the intergenerational digital communication issues portrayed in “Adolescence.” European users generated the largest proportion of relevant posts (35%), with substantial representation from Asia (25%) and North America (20%). This global engagement suggests that the series addressed communication challenges that transcend cultural boundaries, though the specific manifestations and interpretations varied by region. European posts more frequently referenced educational applications of the series, while Asian posts (particularly from Indonesia and Taiwan) more often discussed specific digital symbols and their cultural contexts.

**Table 1:** Geographic distribution of X discussions.

Region	Number of posts	Percentage
Europe	7	35%
Asia	5	25%
North America	4	20%
South America	2	10%
Australia/Oceania	1	5%
Unspecified	1	5%
Total	20	100%

Analysis of engagement metrics revealed significant variation in how different posts resonated with X users. As detailed in Table 2, the original post that generated the highest engagement (511 total interactions) discussed the educational application of “Adolescence” in UK schools as a prevention tool against online radicalization, contrasting this with the absence of similar initiatives in France. This post has an exceptionally high engagement level (accounting for 83% of all interactions in the dataset), indicating resonance with concerns about practical applications of the series’ insights. The next most engaged posts addressed actor performance (12 interactions) and mental health implications (6 interactions), suggesting a secondary interest in both the artistic execution of the series and its psychological insights.

**Table 2:** Most engaged original posts by interaction count.

Post content	Total interactions	Primary theme
“Educational institutions in one European country use this series for prevention programs in secondary schools, while another country implements no similar initiatives. Online content creators promoting certain ideologies face no legal consequences”	511	Educational use of series
“Political figure simultaneously praises series addressing youth issues while questioning rising mental health problems among young people, overlooking that current generation experienced unprecedented digital childhood”	6	Mental health implications
“Fifteen-year-old lead actor could become youngest Emmy nominee in history for performance in this series. Good morning to fellow viewers”	12	Actor performance

Thematic analysis of the X content revealed several recurring themes in discussions of the series’ digital communication. The most prevalent theme in 27% of posts centered on the series’ educational value in highlighting the dangers of digital communication. These posts framed “Adolescence” as an essential teaching tool for parents, educators, and young people, with several explicitly advocating its institutional use in educational contexts. The second most common theme (23% of posts) focused on the series’ portrayal of online radicalization, discussing how communication barriers across generations facilitated the protagonist’s undetected descent into extremism.

Parental responsibility for understanding digital culture was the third central theme, appearing in 18% of posts. These discussions emphasized that parents must adapt to evolving digital communication rather than expecting young people to conform to older communication norms. Many posts (15%) referenced specific scenes that depicted miscommunication, analyzing how these moments illustrated broader communication challenges. The remaining posts were divided between mentions of similar real-world cases (12%) and other themes (5%), including technical aspects of the production.

When examining specific digital symbol categories mentioned in posts, emoji misinterpretation emerged as the most frequently discussed (35% of posts), followed by slang/language barriers (28%) and meme literacy (17%). This distribution aligns with the three primary categories of misunderstanding identified in the series’s content analysis, suggesting that viewers were particularly attentive to these aspects of the narrative. Many posts included specific examples from the series, with several quoting or paraphrasing dialogue from key scenes to illustrate their points about intergenerational communication challenges.

## 4 Discussion

Our comprehensive content analysis of “Adolescence” and examination of X discussions reveal significant insights into gaps in intergenerational digital communication symbol interpretation. These insights illuminate patterns of misinterpretation, their consequences, and potential educational and practical implications for addressing these communication challenges.

### 4.1 Patterns of digital symbol misinterpretation

Our analysis identifies three primary categories of digital symbol misinterpretation that consistently appeared in both the series’ narrative and X discussions. These patterns reveal fundamental differences in how various generations approach and interpret digital communication, with potentially serious implications for intergenerational understanding.

The first and most frequently discussed pattern contrasts contextual and literal uses of emoji. The data from our content analysis and X discussions confirm previous research suggesting fundamental generational differences in emoji interpretation (Capobianco, 2022; Mutual, Physicians, 2023). Older generations predominantly employ emojis as straightforward emotional indicators that directly represent their intended meaning. At the same time, younger users use them ironically, hyperbolically, or with meanings that deliberately subvert their original intent. One particularly insightful X user noted: “The skull emoji scene in Adolescence perfectly captures how parents and kids speak entirely different languages online.” This observation refers to a pivotal scene in episode four in which this misinterpretation had narrative consequences.

This pattern extends beyond simple misunderstanding to potentially dangerous misinterpretation with profound implications. In “Adolescence,” Jamie’s enthusiastic use of the skull emoji (💀) in responding to violent content was interpreted by his parents as typical teenage humor, when it signified profound positive engagement with harmful ideologies. The parents understood the emojis’ expressive function but failed to critically examine what their son found compelling, a subtler but equally dangerous form of communication failure. This fictional representation parallels research findings by Mihăilescu (2024) documents how emoji misinterpretation can lead to significant communication failures between generations in real-world contexts.

In the series, Jamie’s use of terms associated with misogynistic online communities (“based,” “redpilled,” “normie”) went unrecognized by adults until they escalated to explicit threats, highlighting how slang can obscure concerning ideological content from those unfamiliar with specific online subcultures. This portrayal reflects McCulloch (2022) research on how rapidly evolving internet language creates generational linguistic divides that exceed typical generational language differences in speed and complexity.

**Figure 2:**
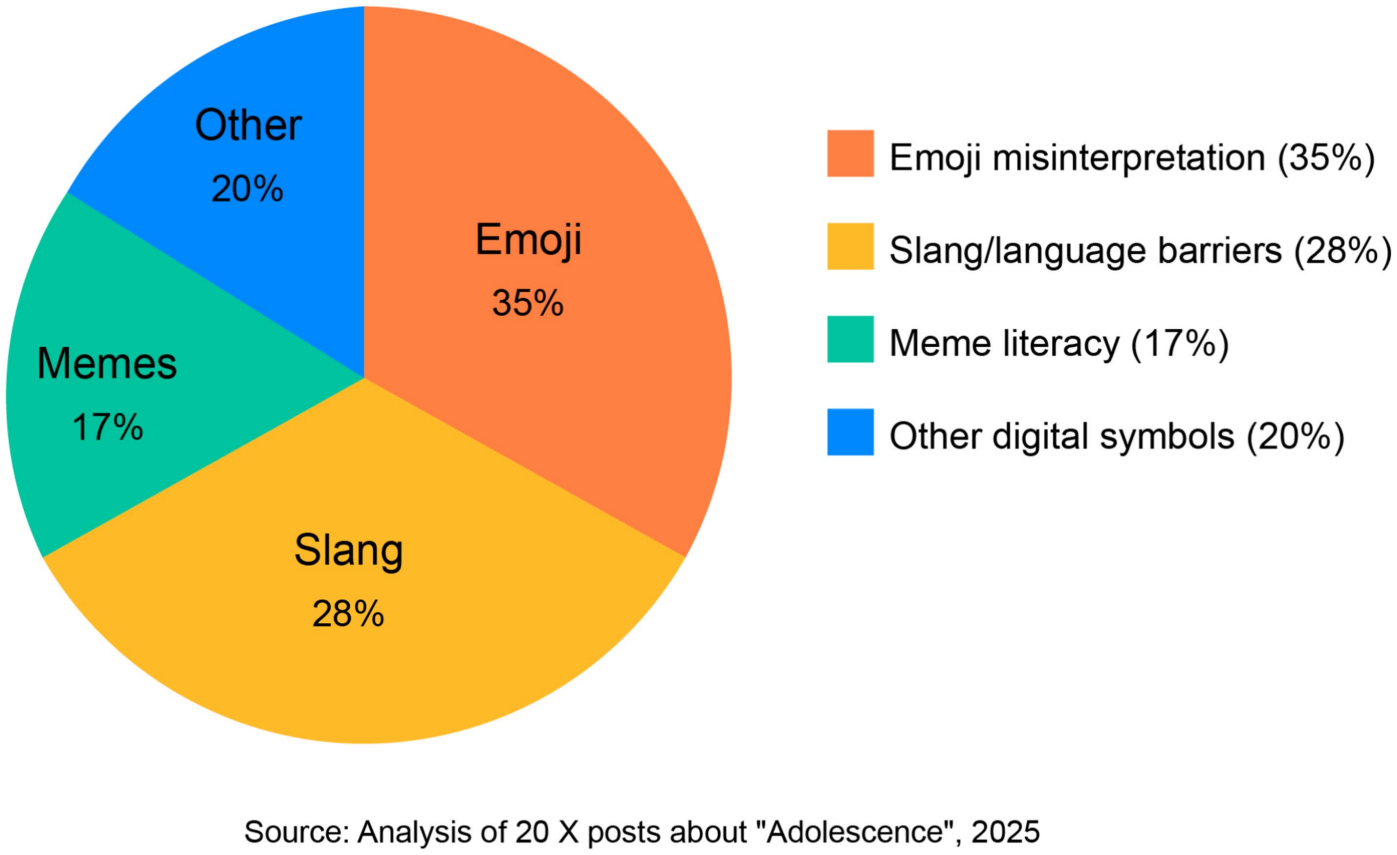
Digital symbol categories mentioned in X discussions.

The third pattern identifies generation-specific cultural references in memes as the most complex form of digital communication, as they require shared cultural knowledge for accurate interpretation. Our content analysis revealed five distinct scenes in “Adolescence” in which Jamie shared memes referencing problematic ideologies, which his parents viewed as harmless because of their lack of familiarity with the referential context. The complexity of meme interpretation emerged in 17% of the analyzed X posts, as shown in our analysis of digital symbol categories mentioned in discussions (see Figure 2).

X users frequently commented on this aspect of the series, with one particularly representative viewer noting: “The most realistic part of Adolescence is how the parents completely miss the red flags in their son’s memes because they do not understand the references.” This observation aligns with Shifman (2023) research documenting how memes function as a cultural literacy test, with meaning often accessible only to those immersed in specific online communities and contexts.

### 4.2 Consequences of digital symbol misinterpretation

Our findings suggest that these misinterpretations have three potential consequences, ranging from interpersonal challenges to institutional failures with potentially severe outcomes.

At the most fundamental level, the inability to comprehend digital communication can erode parent–child relationships. The “Adolescence” narrative and X’s discussions about it portrayed how parents who cannot understand their children’s digital expression often feel increasingly disconnected or unable to provide practical guidance. Simultaneously, children often perceive this disconnection as a lack of interest or dismissal of their cultural reality. This mutual misunderstanding can create a cycle of communication breakdown that widens rather than narrows over time.

Several emotionally resonant scenes in “Adolescence” depicted the deterioration of this relationship, showing how Jamie’s parents’ inability to understand his online communication contributed to his sense of isolation and vulnerability to external influences. This fictional portrayal parallels Livingstone and Blum-Ross (2023) research documenting how misunderstandings in digital communication regularly contribute to family conflict and parental anxiety about children’s online activities.

A more serious consequence involves failure to recognize warning signs of concerning behavior embedded in digital communication. In both the series narrative and X discussions about it, parents’ and authorities’ inability to interpret digital symbols correctly led to missed opportunities for intervention before harmful actions occurred. The series depicted this progression explicitly, showing how misinterpretation of Jamie’s digital communication directly contributed to adults’ failure to recognize his radicalization pathway until it culminated in violence.

The educational implications of this aspect received particular attention in X discussions, with the most engaged post in our dataset (511 interactions) specifically addressing how UK schools were using the series as a prevention tool, contrasting this with a perceived lack of similar initiatives in France (see Table 2). This exceptional engagement level suggests resonance with concerns about the practical applications of the series’ insights into early intervention for problematic online behavior.

The most severe potential consequence involves misinterpreting digital evidence in legal or institutional contexts. Several X posts specifically mentioned the series’ portrayal of how Jamie’s digital communications were initially misinterpreted by authorities investigating the crime, with some expressions being taken as evidence of premeditation when a contextual understanding would have suggested otherwise. This aspect of the narrative highlighted how misinterpretation can affect prevention and responses to harmful actions after they occur.

This fictional representation parallels Howry et al. (2023) the documentation of real-world legal cases in which emoji interpretation has significantly influenced case outcomes, prompting some jurisdictions to develop specialized training for legal professionals in interpreting digital communication. The series thus draws attention to an emerging challenge in institutional responses to evidence from digital communication.

### 4.3 Educational and practical implications

**Figure 3:**
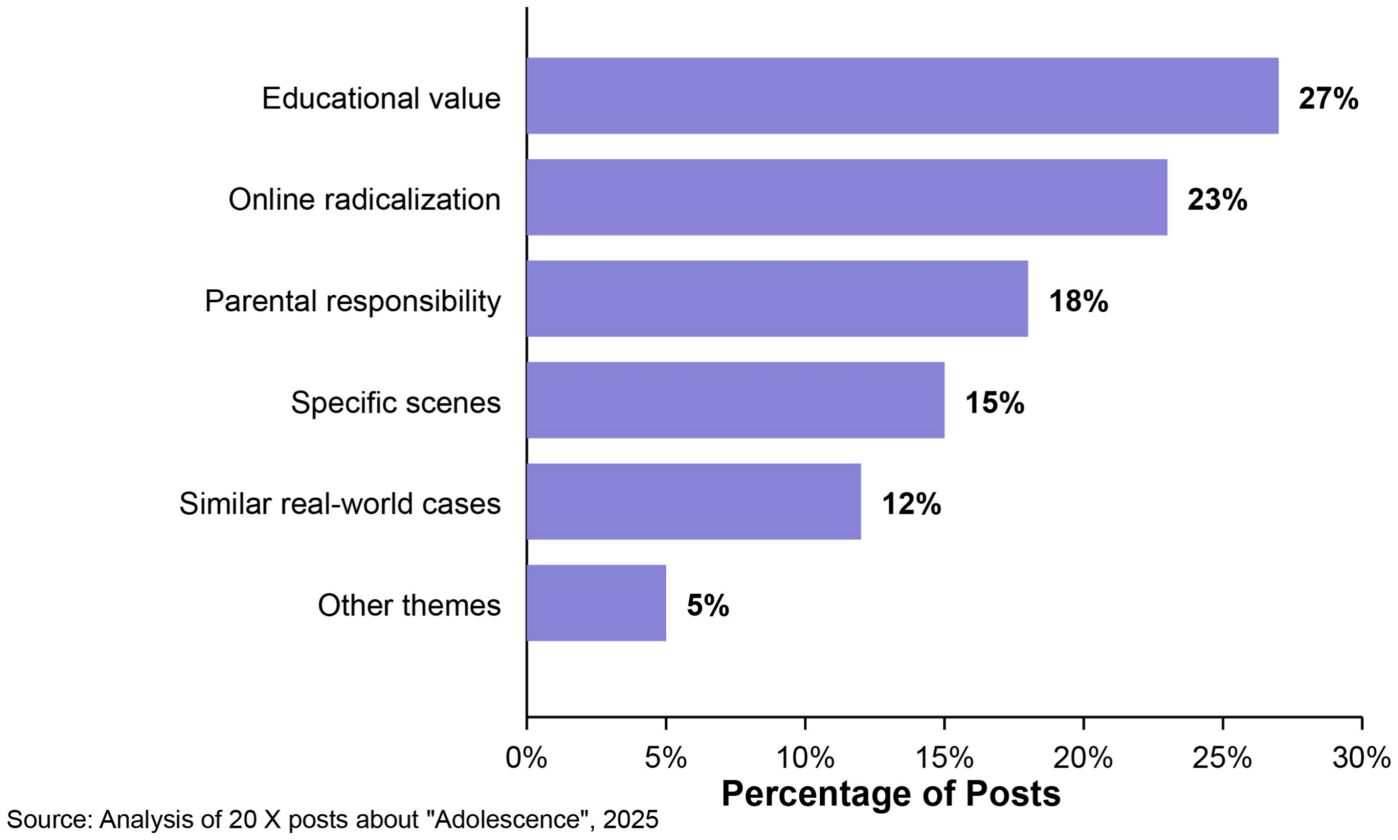
Central themes in X discussions about “Adolescence.”

While this research does not propose or evaluate specific prevention programs, our findings regarding the patterns and consequences of digital symbol misinterpretation provide a foundation for identifying where prevention efforts might be productively targeted. The significant engagement with X posts discussing the educational applications of “Adolescence” suggests widespread recognition of both the importance of these issues and the need for interventions that address them. As shown in our thematic analysis and illustrated in Figure 3, educational value was the most prevalent theme in X discussions (27% of posts), followed by discussions of the portrayal of online radicalization (23%) and parental responsibility (18%). This distribution highlights the educational focus of public discourse about the series.

Based on our identification of three primary categories of misinterpretation (emoji, slang, and memes) and the documented consequences of these communication failures, we can identify several directions for future prevention-focused research and program development. These findings indicate several potential directions for addressing intergenerational challenges in digital communication. First, there is substantial demand for structured educational programs to improve digital communication literacy across generations. Rather than focusing exclusively on teaching older generations about youth digital culture or teaching younger users about traditional communication norms, such programs would ideally foster mutual understanding and appreciation of different communication approaches.

Second, our findings suggest the need for continuously updated resources to help parents, educators, and other adults stay current with rapidly evolving digital symbols. Given the rapid pace at which digital communication norms evolve, static educational materials quickly become outdated. More effective resources include digital platforms that track evolving usage patterns and provide regular updates on emerging trends, symbols, and their contextual meanings. For example, an emerging genre of educational content on TikTok features educators explaining current slang and communication trends being used in classrooms, making this information accessible to parents and other adults in the same formats young people use (see @mr_lindsay_sped and similar accounts). One representative video from this genre defines terms like “sigma,” “rizz,” and “no cap” with clear explanations: “‘Sigma’ refers to someone who operates independently of social hierarchy; ‘rizz’ means charm or ability to attract romantic interest; ‘no cap’ means ‘no lie’ or ‘for real’” (mr_lindsay_sped, 2025). These dynamic, platform-native resources represent a more sustainable approach than traditional printed glossaries, which become outdated before publication.

Third, the series’s portrayal of institutional misinterpretation and associated X discussions underscores the importance of specialized training for legal and institutional authorities to interpret digital communication accurately. The highly contextual nature of digital symbols, particularly in youth communication, creates potential for significant misunderstanding in contexts where interpretation may have serious consequences, such as criminal investigations, educational disciplinary proceedings, or mental health assessments.

Finally, the series and X discussions emphasize the value of open dialogue between generations about differing interpretations of digital symbols. Rather than treating differences in digital communication as merely technical challenges to be overcome through education, this approach recognizes them as cultural differences that require mutual respect and engagement. Several X users praised “Adolescence” for initiating such conversations within their families, suggesting that artistic representations of these issues can bridge intergenerational understanding.

## 5 Conclusion

This study’s primary contribution lies in identifying and characterizing the specific communicative mechanisms through which intergenerational misunderstandings of digital symbols create environments where concerning behaviors can develop undetected. We do not propose or evaluate prevention interventions; rather, we provide foundational analysis that maps the problem space and its underlying mechanisms to inform subsequent intervention development. Our findings establish what needs to be addressed (specific types of symbol misinterpretation), where these issues manifest (contexts and communication channels), and the consequences of these failures (ranging from relationship erosion to missed detection of serious risk behaviors). This descriptive and analytical groundwork is essential for future prevention research, which can build upon our identification of these communicative “blind spots” to design, implement, and evaluate targeted interventions.

Our findings substantiate and expand existing research on differences in digital symbol interpretation across generations, illuminating three key categories of misinterpretation that create particularly significant barriers to intergenerational understanding. The first concerns the contrast between contextual and literal uses of emoji, with younger generations employing emoji in ways that often remain opaque to older users, not because the symbols’ expressive functions are misunderstood, but because adults may fail to examine the content that elicits emotional reactions critically. For example, recognizing that a skull emoji expresses an extreme positive reaction is only valuable if one also investigates what is being reacted to so enthusiastically. The second category encompasses rapidly evolving digital slang that functions as coded language, with expressions gaining and losing specific contextual meanings faster than those outside youth communities can track. The third category involves generation-specific cultural references in memes, which require shared cultural knowledge and familiarity with specific online contexts for accurate interpretation.

The representation of these communication gaps in “Adolescence” portrays them not merely as minor irritations but as potentially serious barriers with significant consequences. Our analysis of both the series content and X discussions reveals three potential levels of impact from these misinterpretations. At the interpersonal level, they can erode family relationships and create cycles of misunderstanding that widen rather than narrow over time. More seriously, they can lead to missed intervention opportunities when adults fail to recognize warning signs embedded in digital communication that uses unfamiliar symbols or references. Most severely, they can lead to institutional misinterpretation with serious consequences in contexts such as legal proceedings, educational disciplinary actions, or mental health assessments.

An important consideration for both research and practical intervention involves recognizing the diversity of generational configurations in parent–child relationships. Not all parents or authority figures are from the same older generation; some parents of adolescents are themselves from Generation Z (particularly older Gen Z members born 1997–2002), while others may be Millennials, Generation X, or even Baby Boomers. This generational diversity has significant implications for digital communication gaps and potential interventions. Younger parents, particularly those from Generation Z, may have substantially smaller digital communication gaps with their children compared to older parents, having grown up with many of the same platforms and communication norms. This suggests that intervention strategies should be differentiated by parental generation, with older parents potentially requiring more comprehensive digital literacy support.

In comparison, younger parents may benefit more from guidance on specific emerging trends and platforms that postdate their own adolescence. Additionally, younger parents may serve as valuable resources for developing and delivering educational content for older parents through peer-mentorship programs or intergenerational learning communities. Future research should examine how parental generation moderates the digital communication gap and whether younger parents’ familiarity with digital culture is associated with more effective recognition of warning signs in their children’s online behavior.

The significant engagement with educational themes in X discussions about “Adolescence,” particularly the exceptionally high interaction count (511 interactions) for the post discussing educational applications in UK schools, suggests widespread recognition of the real-world relevance of these issues. This aligns with the sentiment distribution shown in Figure 1, which indicates a predominantly neutral-to-positive orientation toward discussions of these themes, suggesting receptivity to educational approaches rather than dismissive or polarized reactions.

Our research contributes to the literature on digital literacy and intergenerational communication in several significant ways. First, it grounds theoretical concerns about intergenerational digital communication gaps in a specific cultural case study that has garnered global attention and resonance. Second, it employs a mixed-methods approach that examines fictional representation and real-world discourse, providing insight into how these issues are portrayed and received. Third, it identifies specific categories of digital symbol misinterpretation that recur across both narrative portrayals and public discussions, suggesting focal points for educational intervention.

Perhaps most importantly, this research highlights the mutual nature of intergenerational digital communication challenges. Rather than positioning these issues as simply a matter of older generations needing to “catch up” to youth digital culture or younger users needing to conform to traditional communication norms, both the series portrayal and X discussions emphasize the need for mutual understanding and adaptation. As one X user noted, “The tragedy in Adolescence is not just that parents do not understand kids’ language, nor do they realize they are speaking different languages until it is too late.”

The practical implications of these findings extend across multiple domains. In family contexts, they suggest the value of open dialogue about differences in digital communication and the regular sharing of digital experiences across generations. In educational settings, they indicate the need for curriculum development that addresses digital symbol literacy for both students and educators, emphasizing the contextual nature of digital communication rather than simply teaching specific symbols or terms that are likely to evolve. In institutional contexts, particularly those involving the assessment or evaluation of digital communication evidence, they highlight the importance of specialized training and consultation with experts in contemporary youth digital culture.

### 5.1 Limitations and future research directions

While this study provides valuable insights, it has several important limitations that suggest directions for future research. First, our X data collection was necessarily limited to publicly available posts. It may not represent the full spectrum of discussions about the series, particularly in more private online spaces or through other communication channels. The relatively small sample size of 20 posts that substantially engaged with the digital communication themes, while providing rich qualitative insights, limits statistical generalizability.

Second, our focus on a single case study, albeit a globally significant one that deliberately addresses intergenerational digital communication, limits the breadth of our analysis. “Adolescence” presents a theatrical portrayal of these issues, which may not reflect the more common, everyday manifestations of these communication challenges. Additional research examining less extreme but more typical intergenerational digital communication scenarios would complement our findings.

Third, the rapidly evolving nature of digital communication symbols means that specific examples identified in both the series and X discussions will inevitably become dated, even as the underlying patterns of intergenerational misunderstanding persist. This temporal limitation is inherent to research on digital communication and underscores the need for ongoing investigation rather than a one-time analysis.

Future research should expand on these findings in several directions. Cross-cultural comparative studies would be particularly valuable for understanding how intergenerational digital communication gaps manifest differently across cultural contexts with varying family structures, patterns of technological adoption, and generational relationships. Longitudinal research tracking how these communication gaps evolve could provide insight into whether technological familiarity eventually bridges these divides or whether fundamental differences in approach to digital communication persist regardless of technical proficiency.

Most critically, intervention-focused research is needed to build on our foundational identification of communicative mechanisms and to develop, implement, and evaluate prevention programs that bridge these specific communication gaps. Our research has identified what (types of misinterpretation), where (contexts in which they occur), and why (underlying mechanisms) of intergenerational misunderstandings of digital symbols. The next essential step is intervention research that addresses the how (effective strategies for prevention and remediation). Such research should examine the effectiveness of various approaches, ranging from technology-focused education that teaches specific symbols and their meanings to more communication-focused programs that emphasize mutual understanding and intergenerational dialogue. Additionally, intervention studies should test whether addressing the specific categories of misinterpretation we have identified (emoji interpretation, slang comprehension, and meme literacy) through targeted educational programs reduces the documented negative consequences (relationship erosion, missed warning signs, and evidence misinterpretation).

Finally, institutional research exploring how misinterpretation of digital communication affects specific contexts, such as education, mental health services, and legal proceedings, would help identify where specialized training or protocol development is most urgently needed.

The rapidly evolving nature of digital communication symbols necessitates ongoing research to track new developments and their implications for intergenerational understanding. As “Adolescence” powerfully demonstrates through its narrative. As the global X engagement with the series confirms, these are not merely academic concerns but issues with potential real-world consequences for individuals, families, and communities navigating an increasingly digitally mediated world.

## Data Availability

The raw data supporting the conclusions of this article will be made available by the authors, without undue reservation.
